# Practical Issues of Medical Experts in Assessing Persons With Mental Illness Asking for Assisted Dying in Switzerland

**DOI:** 10.3389/fpsyt.2022.909194

**Published:** 2022-07-07

**Authors:** Henning Hachtel, Daniel Häring, Tanya Kochuparackal, Marc Graf, Tobias Vogel

**Affiliations:** ^1^Clinic for Forensics, University Psychiatric Clinic Basel, Basel, Switzerland; ^2^Independent Researcher, Basel, Switzerland

**Keywords:** capacity of judgement, Code of Professional Conduct, medical-ethical guidelines, Swiss criminal and medical law, multidisciplinary assessments, end-of-life-wish, severe suffering

## Abstract

With about 65,000 deaths per year in Switzerland, about 1,000 assisted suicides of Swiss citizens are carried out with the help of assisted dying organizations per year. Assisted suicide, which is carried out without selfish motives on the side of the helping person, only remains unpunished if there is a free will decision by the person willing to die who has the capacity of judgement and to act independently. While this is usually accepted as an option for somatically terminally ill patients in society at large, this procedure is controversial for psychiatrically ill patients. In Switzerland the topic of assisted dying is highly debated between medical professionals. In 2018, the Swiss Academy of Medical Sciences (SAMS) put revised guidelines into force, which are in discrepancy to the current rules of the Swiss Medical Association (FMH). This article gives an overview of the past and current development of the Code of Professional Conduct and medical-ethical guidelines as well as current Swiss criminal and medical law on this topic. Practical implications for the assessment of assessing persons with mental illness in this circumstances are discussed. It is to be concluded, that persons with a mental illness seem to face extra obstacles in relation with somatically ill persons as the assessment of the prerequisites comprises additional requirements. Among other issues there is an urgent need for the elaboration of contents to be assessed and standards of procedures. The procedures and guidelines to be elaborated should be scientifically accompanied in order to gain a more reliable basis for decision-making. Multidisciplinary assessments would help to avoid biases and blind spots of a mono-disciplinary assessments. In addition, even in the case of mentally ill people, their right to self-determined suicide should not be restricted by excessive hurdles in the assessment process. Lastly, reliable funding should be secured, as it is otherwise to be expected that the complex assessment of prerequisites through multi-professional-teams or just one assessor cannot be sustained. The exercise of fundamental rights must be possible for all persons to the same extent, regardless of their financial resources.

## Introduction

Assisted dying is a social issue preoccupying legislations, jurisdiction, and politics worldwide. The term “assisted dying” covers very different forms of assistance in dying. Especially voluntary euthanasia, i.e., to end a person's life at her/his own express request, is to be distinguished from (physician-supported) assisted suicide, i.e., to help a person, at her/his own express request, to end her/his life e.g., by providing the means such as prescription or medications. The main difference between the two concepts hence is, that in assisted suicide the patient takes the final action while in voluntary euthanasia this action is performed by another person (1). Misuse of the term euthanasia cumulated during the reign of German National Socialists in the murder of people with disabilities, mental disorders, low social status, or gay people (2). Assisted suicide on the other hand is a term less burdened with associations of historical events and relations like the term euthanasia more to murder (1).

Assisted Suicide can be legally practiced in the Netherlands, Belgium, Luxembourg, Switzerland, Spain, Germany, Colombia, and Canada, as well as in nine states and the district of Columbia within the United States, and the Australian state of Victoria (3). In Italy, legislation moved a step closer to legalizing a form of euthanasia through a vote of members of parliament, who voted in favor of a new law that would allow “voluntary medically assisted death” for ill patients in March 2022. The final vote of Italy's Senate is still needed before it can be passed into law. In Germany, amongst other groups, the professional society of psychiatrists and neurologists published a statement concerning points for a possible new regulation of assisted suicide (4) as the German Federal Constitutional Court's ruling on the 26th of February 2020 declared the pre-existing ban on assisted suicide null and void. In Austria, the judgment of the Constitutional Court on the 11st of December 2020 stated that the absolute prohibition of any assisted suicide is unconstitutional. After the Austrian Dispositions of Dying Act, which entered into force as of January the 1st 2022, a detailed listing of scope, definitions, and prerequisites were published in a Federal Law Gazette (5). The debate today is mainly about the legality of assisted dying in all its forms as such and whether such assistance is only possible when people are at the end of life. In the Netherlands, Belgium, Luxembourg, and Switzerland (3) and recently Austria, assisted suicide is not restricted to patients at the end of life stage.

Since 1985, physician-supported assisted suicides have been offered by specific organizations, such as the two Swiss EXIT non-profit associations, and case numbers of assisted suicide have risen continuously, among Swiss residents but also residents of other countries (mainly Germany, UK and France), who travel to Switzerland because they want professional accompaniment in suicide, which they do not receive in their home countries Most commonly malignancies (among Swiss residents) or neurological disorders (among residents of other countries), followed directly by age-related functional impairments were named as cause in this context (6). The following content refers to the situation in Switzerland.

## Ethical and Medico-Legal Guidelines

In December 2006, the Swiss Federal Supreme Court had to decide for the first time about the desire to receive access to the means to suicide—i.e., 15 g of pentobarbital—for the purpose of suicide of persons with a mental illness (BGE 133 I 58 ff.). The court held that an incurable, permanent, severe mental impairment, similar to a somatic impairment may constitute a condition that makes the patient's life no longer worth living in the long term. However, it needs to be distinguished between the desire to die due to a treatable mental disorder, which calls for treatment, and the desire to die based on a self-determined, well-reflected and durable decision of a person capable of judgment. If the wish to die bases on an autonomous decision which takes into account the overall situation which is to be determined by an in-depth expert (psychiatric) opinion, assisted suicide may also be granted to mentally ill persons under certain circumstances (7). As part of the regulation of assisted suicide the Federal Supreme Court's judgement defined as requirements the personal examination and assessment of capacity of judgment by the physician, an informative interview, a correct diagnosis and indication. Furthermore, assisted suicide of mentally ill persons should only be possible with the utmost restraint and supported by a professional opinion (BGE 133 I 58 ff.).

As a result, (medical supported) assisted suicide is also permitted for persons with mental illness according to current case law in Switzerland. Assisted suicide, which is carried out without selfish motives on the side of the helping person, is legal if there is a free will decision by the person willing to die who has capacity of judgement and to act independently. According to figures from the assisted dying organization Exit 2018, around 1.5% of all deaths of Swiss citizens involve assisted suicide by an assisted dying organization. With about 65,000 deaths per year, this means about 1,000 assisted suicides of Swiss citizens by all assisted dying organizations per year. The Swiss Federal Statistical Office also reports on the frequency of assisted suicides, but only up to 2014: “From 1995 to 2003, the absolute number of suicides decreased significantly, since then it has been roughly constant, while cases of assisted suicide have increased significantly, especially since 2008. In 2014, for every 7 cases of suicide, there were 5 cases of assisted suicide” (8). Other literature state as a trend for 2016 that the numbers of cases of assisted suicide now almost match those of conventional suicide (6).

Accordingly, it can be assumed, that assisted dying is a relevant social phenomenon. Several polls have so far shown that the Swiss people want a liberal regime regarding the possibility of assisted dying. Also a representative national survey in May 2010 of around 1,500 Swiss people on their attitudes toward assisted suicide showed a liberal attitude in general, but a more skeptical attitude toward extending assisted dying to people with severe mental disorders and to older, “tired of life” people without physical complaints (9). A survey of German-speaking psychiatrists in Switzerland reported that the majority of respondents would not support a request for assisted suicide in cases of severe mental disorder (10). This is in contrast to current concepts in the care of people with severe mental impairments, which emphasize empowerment, autonomous decision-making and the assumption of “positive risks” (i.e., looking beyond the potential physical effects of risk and considering the mental aspects of risk, such as the effects on wellbeing or self-identity if a person is unable to do something that is important to them). One could argue therefore, that people with severe mental impairments are encouraged to live their lives in a self-determined way and dare to live despite any disabilities (11).

In contrast to the treatments for somatic diseases, the involuntary treatment of disorders is an ongoing issue in psychiatry throughout medical history. Involuntary treatment denotes medical treatment given without informed consent from the patient. Literature suggests that the use of involuntary methods varies across Europe and to obtain clinical data about it is difficult (12). The association of a more frequent involuntary aspect in psychiatric treatments and a higher perceived stigma of psychiatric diseases (13) might help to explain the difference in regarding the possibility of assisted dying in mental and somatic disorders in the public. From a legal perspective, it should be noted that there is no compulsory treatment in Switzerland. This also applies to mentally ill persons in case of full capacity of judgement. Also, in case of impaired judgement in relation to the mental disorder there are high hurdles for compulsory treatments of mentally ill persons. Thus, there is a tension between the psychiatrically sometimes necessary compulsory treatment and the right of self-determination of mentally ill persons. In any case, the extensive interpretation of compulsory treatment must not lead to mentally ill persons no longer being able to exercise their fundamental right to a self-determined death.

In Switzerland the topic of assisted dying is highly debated between medical professionals. The Swiss Academy of Medical Sciences (SAMS) first published guidelines on assisted suicide in 1976. The latest revision in 2018 included a reorientation with a broad focus (including assistance in conducting conversations, concept of health advance planning) and a new subchapter on assisted suicide (14). In the following public consultation and discussion in the Swiss Medical Association FMH all innovations were expressly welcomed with the exception of assisted suicide. It was stated that the term “unbearable suffering” was too vague and subjective for a valid assessment which would possibly lead to an inflationary expansion of requests for assisted dying. As a consequence, the majority of the FMH rejected the inclusion of the revised guidelines into the Code of Professional Conduct and the former version of 2004 is still valid under the FMH perspective. Although, the SAMS put the revised 2018 guidelines into force which leads to a discrepancy between the FMH-rules and the SAMS-rules. In the meantime the SAMS initiated an approval process with the final goal of including a newer version of 2021 guidelines in the Code of Professional Conduct (instead of the old version of 2004) in 2022. The new version was no longer discussed in public, but submitted directly to the FMH by the SAMS without consultation with the physicians. The FMH approved the new version in May 2022, again without consulting the members (doctors). The current version states among other things, four necessary prerequisites to be eligible for medical assisted suicide as ethically responsible in individual cases: (1) Capacity of judgement in relation to assisted suicide (no assisted suicide may be performed if the desire to die is a current symptom of a mental disorder); (2) Autonomous will; (3) Presence of severe suffering (limitations classified as serious by the person with functional limitations must be substantiated by a corresponding diagnosis and prognosis); (4) Consideration of alternatives. As part of the established practice after the initial contact of the person asking for assisted suicide with a general practitioner or assisted dying organization, a first assessment and expert opinion assessing the stated prerequisites is following in short order before further steps are initiated (15). However, it is important to highlight that only the first two prerequisites have a legal basis (capacity of judgement and autonomous will); the other two (severe suffering and consideration of alternatives) are not found in the law or the relevant regulations; they are from a mere legal perspective non-binding requirements, issued by a private organization. The Swiss Federal Supreme Court also stated in a new decision at the end of 2021 that the rules of the SAMS and the FMH were not binding rules of purely private origin (BGer 6B_646/2020, E. 1.6). As long as there are no legally binding guidelines, each expert must form his or her professional opinion according to his or her best professional knowledge and conscience, free of any guidelines.

## Practical Implications

In this respect the growing demand of assisted suicide can be labeled a social reality. On this issue the current Code of Professional Conduct is more restrictive than the proposed medical-ethical guidelines of the SAMS. Additionally to consider is that according to current Swiss criminal and medical law there is no prerequisite that for assisted suicide therapeutic options of an illness are exhausted. The law does not recognize compulsory treatment. Thus, no additional hurdles should be created for mentally ill persons that do not exist for somatically ill persons also. If, for example, there is no compulsory therapy for somatically ill persons, there should also be no compulsory therapy for mentally ill persons. As a result, the assessment of persons with mental illness asking for assisted suicide is still in a field of tension of uncertain professional law and possible professional consequences for willing expert assessors. This extra obstacle lowers the chances of persons with mental illness finding a professional willing and capable of assessing his/her wish for an assisted suicide and establishing whether (or not) the necessary prerequisites are fulfilled. Noteworthy in this context is the current Swiss Federal Court jurisprudence: the Federal Supreme Court has, as mentioned already, ruled several times that in this context the rules of the SAMS and the FMH are not binding rules of purely private origin (BGer 6B_646/2020, E. 1.6). The duties of a physician are governed by the law, not by any guidelines issued by private organizations.

The assessment of the capacity of judgement by expert assessors is a standard procedure which normally includes in Switzerland the evaluation of cognition, valuation capability, formation of will and willpower (16). The respective guidelines of the SAMS recommend a thorough documentation of the results of the assessment if these are made in connection with the fundamental wish of dying. Careful documentation is also important in case of supervisory or criminal proceedings. The SAMS Medical-ethical guidelines of 2004 are still valid for professional law and declares that the end-of-life-wish is well weighed and permanent, including the evaluation of these therapeutic possibilities against the background of their personal experiences and value convictions (17). The majority of the 2004 prerequisites are not found in the law either. Nevertheless, these criteria are of some importance in practice, although there are various open questions about them.

There is, e.g., no statement to be found after which time period an end-of-life-wish is to be considered “permanent,” especially regarding mental illnesses. Also the assessment of “severe suffering” on contains room for interpretation when there are no points of reference using algorithms or statistical tools. In palliative care medicine, the vaguely defined role of the patient was partly held responsible for the considerably varying guidelines how to determine “intolerable suffering” for the application of sedation therapy (18). As possible indications in these guidelines were designated psychological and existential suffering associated with a clear lack of precise definitions of the respective terms (18). In analogy to a forensic approach in criminal law the expert assessor could revert to a dimensional approach of assessing impairments of a given disorder in relation to the most possible expression of symptoms of severe disorders like schizophrenia or dementia (19). Whilst this produces an approximation of the seriousness of functional limitations, the severity of subjective suffering can only be approached by describing the practical life impact of the mental illness and the cognitive and emotional reaction of the assessed person thereof. The scope for interpretation follows when classifying the suffering of another person as severe. As this delicate issue is criticisable without hard “guarding rails,” it is also recommendable to document the rationale of the assessment carefully in this regard, too. Vandenberghe (20) proposed in this line a long and elaborate exploration and evaluation process in psychiatric illness, including in cases of non-terminal illness, a committee-based multidisciplinary evaluation before assisted suicide. In this area too, however, it should be pointed out that the Swiss Federal Supreme Court has ruled in a very recent decision that assisted suicide for completely healthy people is also legal in principle (BGer 6B_646/2020). The requirement of “severe suffering” does not exist in law. Against this background, no excessive requirements may be made in the case of psychiatric illnesses either.

The needed resources for this model are substantial and put a special focus on mental in relation to somatic illnesses. This argumentation can be followed as many mental disorders are characterized by variable courses and may be influenced to a substantial degree by social conditions (17). A possible approach in the assessment of end-of-life-wishes in persons with mental illnesses could be the collaboration of different experts, such as psychiatrists, psychologists or other professions (if necessary) to better cover differing fields of expertise (e.g., psychologists focusing on personality disorders, psychiatrists on schizophrenia spectrum disorders). Also the multidisciplinary proceeding would help to avoid biases and blind spots of a mono-disciplinary assessment. This procedure would help reduce personal beliefs and motivations for action. The until now missing standardization of procedure, content and form in relation to severe suffering and permanence of the wish to die is additionally calling for a multi-person approach before the background of a delicate issue of professional conduct. A further possible benefit would be that in line with the reasoning of Vandenberghe (20) recovery-oriented care could continue in parallel informed by the team of assessors. Possible offers of help and support could then be maximized and as a result social conditions which could be target of change be identified or the immutability of which established. As social conditions change, the end-of-life-wish may change as well—or endure.

In the case of assisted dying organizations after contact with staff, it is a possibility, that at the moment when assisted suicide is open to persons who wish to die, that they not only refrain from “hard” methods but also renounce assisted suicide altogether or postpone it. It can be observed that people who are given the option of a safe and “soft” assisted suicide find new strength for their current situation. This increases the defensibility of assessments made as well in view of possible litigations by e.g., family feuds. Not unmentioned in regards to assisted suicide should be concerns about historical aspects, i.e., the role of psychiatry in the perversion of the concept of euthanasia especially in the German language area during the era of national socialism. The multi-person assessments would also minimize the possibility of simplifying irrevocable decisions about life and death and the consequences thereof. However, despite all the advantages of a multi-person assessment, care must be taken not to complicate the assessment unnecessarily. The Federal Supreme Court has ruled that mentally ill people also have the right to make their own decisions about the end of their lives (BGE 133 I 58 ff.). This right should not be restricted by excessive hurdles in the assessment process. And since a person's capacity of judgement is presumed by law (article 16 Swiss Civil Code), it is finally not a question of assessing whether someone is capable of judgement, but whether there are valid reasons that speak against this presumed capacity. In case of doubt, it must therefore be assumed that the person has capacity of judgement.

Keeping in mind the above-mentioned difficulties, a possible expert has to consider some financial aspects as well: assistance to suicide is prohibited if it is given for selfish motives (Swiss Criminal Code, article 115). Thus, the questions arises if taking considerable time to assess a delicate, possible ambiguous matter is an option for a wider number of professionals, if it is unclear how compensation for this can be structured. However, the absence of selfish motives does not mean one has to act altruistic. From a legal point of view it was stated, that if the compensation is based on the expert's administrative costs and expenses or if customary professional wages or fees are demanded, this is not to be considered as selfish motives within the meaning of art. 115 (7). The expert's financial motives only then become selfish, if professionals claim earnings significantly above the scope of the market norm, without any specific reason to do so. Persons with mental illness asking for assisted suicide on the other hand could be unable to finance an assessment lege artis and would thereof be deprived of the possibility granted to more financially backed peers or would have to resort to assessors without the above mentioned claims to standards. The exercise of fundamental rights must be possible for all persons to the same extent, regardless of their financial resources.

## Conclusions

The wish of assisted suicide has become a social reality for a substantial proportion of the population, including—to a certain extend—persons with a mental illness. Under the current SAMS Medical-ethical guidelines persons with a mental illness seem to face extra obstacles in relation with somatically ill persons as the assessment of the prerequisites comprises additional requirements. One demand therefore must be an ongoing discourse on professional ethics that should not be tabooed. Another urgent need is the elaboration of contents to be assessed and standards of procedures. Preferably, the professional organizations FMH and/or SAMS should initiate a public consultation and discussion with the goal to approve viable guideline for medical practitioners. The procedures and guidelines to be elaborated should be scientifically accompanied in order to gain a more reliable basis for decision-making in the public discussion. Also, the assessment by more than one expert could be included in future guidelines. In addition, even in the case of mentally ill people, their right to self-determined suicide should not be restricted by excessive hurdles in the assessment process. Lastly, the remuneration of working time of professionals should be funded by foundations, government authorities or special insurance companies with the inherent goal that all parts of society can get access to impartial and qualified experts. If funding is not secured, it is to be expected that the complex assessment of prerequisites through multi-professional-teams or just one assessor cannot be sustained.

## Author Contributions

HH, TV, and TK contributed to conception and design of the article. HH wrote the first draft of the manuscript. DH wrote sections of the manuscript. All authors contributed to manuscript revision, read, and approved the submitted version.

## Conflict of Interest

The authors declare that the research was conducted in the absence of any commercial or financial relationships that could be construed as a potential conflict of interest.

## Publisher's Note

All claims expressed in this article are solely those of the authors and do not necessarily represent those of their affiliated organizations, or those of the publisher, the editors and the reviewers. Any product that may be evaluated in this article, or claim that may be made by its manufacturer, is not guaranteed or endorsed by the publisher.
